# Cyr61 promotes CD204 expression and the migration of macrophages via MEK/ERK pathway in esophageal squamous cell carcinoma

**DOI:** 10.1002/cam4.401

**Published:** 2015-01-26

**Authors:** Manabu Shigeoka, Naoki Urakawa, Mari Nishio, Nobuhisa Takase, Soken Utsunomiya, Hiroaki Akiyama, Yoshihiro Kakeji, Takahide Komori, Yu-ichiro Koma, Hiroshi Yokozaki

**Affiliations:** 1 Division of Pathology, Kobe University Graduate School of MedicineKobe, Japan; 2 Division of Oral and Maxillofacial Surgery, Kobe University Graduate School of MedicineKobe, Japan; 3 Division of Gastro-intestinal Surgery, Kobe University Graduate School of MedicineKobe, Japan

**Keywords:** CD204, Cyr61, esophageal cancer, macrophage, tumor microenvironment

## Abstract

Tumor-associated macrophages (TAMs) are known to be involved in the progression of various human malignancies. We previously demonstrated that CD204 was a useful marker for TAMs contributing to the angiogenesis, progression, and prognosis of human esophageal squamous cell carcinoma (ESCC). We also showed that conditioned media of ESCC cell lines induced CD204 expression in THP-1 human monocytic leukemia cells. Here, we performed a cDNA microarray analysis between THP-1 cells stimulated with TPA (macrophage [MΦ]-like THP-1 cells) treated with and without conditioned medium of ESCC cell line to clarify the molecular characteristics of TAMs in ESCC. From the microarray data, we discovered that Cyr61 was induced in CD204-positive-differentiated THP-1 cells (TAM-like THP-1 cells). In the ESCC microenvironment, not only cancer cells but also TAMs expressed Cyr61. Interestingly, the expression levels of Cyr61 showed a significant positive correlation with the number of CD204-positive macrophages in ESCCs by immunohistochemistry. Recombinant human Cyr61 (rhCyr61) promoted cell migration and induced the expression of CD204 along with the activation of the MEK/ERK pathway in MΦ-like THP-1 cells. Pretreatment with a MEK1/2 inhibitor significantly inhibited not only the Cyr61-mediated migration but also the CD204 expression in the MΦ-like THP-1 cells. These results suggest that Cyr61 may contribute to the expression of CD204 and the promotion of cell migration *via* the MEK/ERK pathway in TAMs in the ESCC microenvironment.

## Introduction

Macrophages, the most abundant cancer stromal cells, may contribute to the composition of specific microenvironment for the tumor progression by interacting with cancer cells as well as other stromal cells. Evidence of a correlation between the infiltration of tumor-associated macrophages (TAMs) and poor prognosis in various human malignancies has been accumulating.^1^ TAMs play important roles in tumor progression by facilitating angiogenesis, matrix remodeling, and cancer cell motility and immunosuppression.^1^–^3^ Macrophages are proposed to differentiate into tumor-suppressive (M1) or tumor-supportive (M2) phenotypes in response to various stimuli from the microenvironment.^4^–^6^ Macrophages with the M2 phenotype, characterized by high interleukin (IL)-4, IL-10, and low IL-12 production, are frequently found among TAMs.^2^,^3^ Specific monoclonal antibodies to CD163, a membrane protein belonging to the scavenger-receptor cysteine-rich domain family, and to CD204, a macrophage scavenger receptor, have been developed and were found to be useful for the detection of macrophages with the M2 phenotype.^1^,^2^,^7^

Esophageal cancer is the eighth most common cancer worldwide and is regarded as one of the intractable human cancers, with an estimated 456000 new cases (3% of all cancers) and 0.4 million cancer deaths (5% of all cancer deaths) in 2012.^8^ Although the incidence of adenocarcinoma is increasing in the United States and Western countries, ESCC is still the leading histological subtype in Asian countries including Japan. From the histopathological view point, close interactions of neoplastic squamous cells and stromal components are clear even in the intraepithelial lesions with the characteristic intrapapillary capillary loop alterations that are now good diagnostic clues to ESCC with narrow band imaging endoscopy.^9^ However, the roles of the stromal cells in the carcinogenesis and promotion of ESCCs have not been elucidated.

We recently reported that the number of infiltrating CD204^+^ macrophages in ESCC tissues was significantly associated with microvessel density, depth of tumor invasion, vessel infiltration, lymph node metastasis, clinical stages, and disease-free survival of the patients.^10^ We also demonstrated that conditioned media of five ESCC cell lines (TE-8, TE-9, TE-10, TE-11, and TE-15) induced the mRNA and protein expressions of CD204 along with the mRNA upregulation of *vascular endothelial growth factor A* (*VEGFA*) in THP-1 human monocytic leukemia cells in vitro.^10^ These findings suggested that CD204 was a useful marker for TAMs contributing to the angiogenesis, progression, and prognosis of ESCCs whose specific microenvironment might result in acquiring the macrophages to be CD204^+^ M2 phenotype.

Based on these backgrounds, we conducted a cDNA microarray analysis between THP-1 cells treated with and without conditioned media of ESCC cells to clarify the specific gene expression characteristics of TAMs in ESCC.

## Materials and Methods

### Cell cultures

Five ESCC cell lines (TE-8, TE-9, TE-10, TE-11, and TE-15) were obtained from the RIKEN BioResource Center (Tsukuba, Japan).^11^ The individuality of the TE series ESCC cell lines was confirmed by a short tandem repeat analysis at RIKEN and at the Cell Resource Center for Biomedical Research, Institute of Development, Aging and Cancer, Tohoku University (Sendai, Japan). We purchased the THP-1 human acute monocytic leukemia cell line from the American Type Culture Collection (Mannasas, VA).^12^ We routinely maintained ESCC cell lines and THP-1 cells and prepared the conditioned media of TE series ESCC cell lines (TECMs) as described.^10^,^13^ Mycoplasma was negative in the cell lines by Venor®GeM Classic Mycoplasma Detection kit (Minerva biolabs, Berlin, Germany). To induce macrophage (MΦ)-like differentiation, we also treated THP-1 cells with TPA (Cell Signaling, Danvers, MA) (MΦ-like THP-1 cells) and TECMs for 2 days (TAM-like THP-1 cells) as described.^10^

### cDNA microarray analysis

Total cellular RNA was extracted using the RNeasy Mini Kit (Qiagen, Hilden, Germany). We performed the cDNA microarray using the SurePrint G3 Human GE 8x60K Microarray (Agilent Technologies, Palo Alto, CA). The in vitro transcription, oligonucleotide array hybridization, and scanning were conducted with Takara Bio protocols (TaKaRa Bio, Shiga, Japan). The data have been deposited to the Gene Expression Omnibus repository (GSE59471).

### RT-PCR and quantitative RT-PCR

RT-PCR was carried out as described.^14^ Real-time RT-PCR amplifications of *CD204* and control gene *GAPDH* were performed using the ABI StepOne Real-time PCR system (Applied Biosystems, Foster City, CA). The threshold cycle (Ct) values were determined by plotting the observed fluorescence against the cycle number. We analyzed the Ct values of *cysteine-rich, angiogenic inducer, 61*(*CYR61*), and *CD204* using the comparative Ct method and normalized them to those of *GAPDH*. The relative gene expression levels were estimated using the following formula: relative expression = 2^−(Ct [target gene] − Ct[*GAPDH*])^. Table1 shows the primers we designed according to the previous reports.^15^,^16^

**Table 1:** The primer sets used for RT-PCR and real-time RT-PCR in this study

Gene	Primer sequences
*CYR61*	F: 5′-CTC CCT GTT TTT GGA ATG GA -3′
	R: 5′-TGG TCT TGC TGC ATT TCT TG -3′
*CD204*	F: 5′-CCA GGG ACA TGG GAA TGC AA -3′
	R: 5′-CCA CTG GGA CCT CGA TCT CC -3′
*GAPDH*	F: 5′-ACC ACA GTC CAT GCC ATC AC-3′
	R: 5′-TCC ACC ACC CTG TTG CTG TA-3′

F, forward; R, reverse; PCR, polymerase chain reaction.

### Enzyme-linked immunosorbent assay

We collected and diluted TECMs for the measurement of Cyr61 using the Quantikine ELISA Human Cyr61/CCN1 (R&D Systems, Minneapolis, MN) according to the manufacturer's recommendations. A standard curve was generated for each plate and used to calculate the absolute concentration of Cyr61.

### Tissue samples

A total of 70 sporadic human ESCC tissue samples surgically removed at Kobe University Hospital (Kobe, Japan) were used. The patients were 55 men and 15 women (age range, 54–88 years, mean, 65.7 years). None of the patients received adjuvant chemotherapy or radiotherapy before surgery. Informed consent was obtained from all patients, and the study was approved by the Kobe University Institutional Review Board. All resected specimens were fixed in 10% formalin and embedded in paraffin. Histological and clinicopathological evaluations were performed according to the Japanese Classification of Esophageal Cancer proposed by the Japan Esophageal Society^17^ along with the TNM classification of the Union for International Cancer Control (UICC).^18^

### Immunohistochemistry

We used a modified version of the immunoglobulin enzyme bridge technique with a Linked Streptavidin-Biotin kit (DakoCytomation, Glostrup, Denmark) as described elsewhere.^10^ We used a specific rabbit polyclonal antibody against Cyr61 (H-78, Santa Cruz Biotechnology, Santa Cruz, CA) and mouse monoclonal antibodies to CD204 (SRA-E5, Trans Genic, Kobe, Japan), CD163 (10D6, Novocastra, Newcastle upon Tyne, UK), and CD68 (Kp-1, DAKO) for the primary reaction. After gentle rinsing with 0.05 M Tris-HCl, the sections were incubated with biotinylated goat anti-rabbit or anti-mouse IgG and streptavidin conjugated to HRP. Chromogenic fixation was carried out by immersing the sections in a solution of 3,3′-diaminobenzidine. Sections were counterstained with Mayer's hematoxylin. The macrophage count in the ESCC tissue samples was conducted as described previously.^10^

### Immunofluorescence

For the immunofluorescence examination, formalin-fixed and paraffin-embedded tissue sections were stained with antibodies against Cyr61 (Santa Cruz Biotechnology) and CD204 (Trans Genic). Alexa Fluor 488-conjugated anti-rabbit IgG and Cy3-conjugated anti-mouse IgG (Jackson ImmunoResearch, West Grove, PA) were used as the secondary antibodies. The nuclei were stained with DAPI (Wako, Osaka, Japan). Cyr61- and CD204^+^ cells were observed under a laser-scanning microscope (LSM700; Carl Zeiss, Oberkochen, Germany) and analyzed using the LSM software ZEN 2009 (Carl Zeiss).

### Western blotting

Cells were lysed in a buffer containing 50 mmol/L Tris-HCl (pH 7.4), 125 mmol/L NaCl, 0.1% Triton X-100, and 5 mmol/L ethylenediaminetetraacetic acid or RIPA buffer (Thermo Fischer, San Jose, CA) containing both 1% protease inhibitor and 1% phosphatase inhibitor cocktail (Sigma, St Louis, MO). The resulting lysates were separated on 5–20% SDS polyacrylamide gels, transferred to a membrane with iBlot Gel Transfer Stack (Invitrogen, Carlsbad, CA, USA), and reacted with antibodies against CD204 (Trans Genic), MEK1/2 (Cell Signalling Technology, Beverly, MA), pMEK1/2 (Cell Signalling Technology), ERK1/2 (Cell Signalling Technology), pERK 1/2 (Cell Signalling Technology), and GAPDH (Santa Cruz Biotechnology). After washing, the blots were incubated with HRP-conjugated anti-mouse or anti-rabbit IgGs (GE Healthcare, Little Chalfont Buckinghamshire, UK). Then, the blots were then probed with ImmunoStar Reagents (Wako).

### Transwell assay

The migration assay was performed using 24-well transwells units, each with an 8-*μ*m pore size filter (BD Falcon™; Becton Dickinson, Lincoln Park, NY). Prior to examination, 3 × 10^5^ THP-1 cells were differentiated to macrophage-like cells in the upper inserts. The insert was then exposed to the lower chambers in the presence or absence of 100 ng/mL recombinant human Cyr61 (rhCyr61; R&D Systems). To determine the effect of anti-Cyr61 neutralizing antibody H-78 (Santa Cruz Biotechnology) on the migration of MΦ-like THP-1 cells, we applied 50% TE-10CM in the lower chamber with or without H-78 or control IgG (Santa Cruz Biotechnology). The cells were cultured for 24 h at 37°C in a CO_2_ incubator. The cells remaining in the upper inserts were completely removed with gently swabbing. The number of cells that had migrated through the membrane into lower chambers was counted using Diff-Quik (Sysmex, Kobe, Japan).

### MEK1/2 inhibitor pretreatment

The pretreatment of MΦ-like THP-1 cells with the MEK1/2 inhibitor U0126 (Cell Signalling Technology) was conducted before the rhCyr61 treatment according to the manufacturer's recommendations.

### Statistical analysis

We analyzed the relationships between the clinicopathological features of ESCCs and the immunohistochemical results were analyzed by *χ*^2^ test. The statistical significance of differences in the in vitro assay results was evaluated by paired *t*-test. A *P *< 0.05 was considered significant. Statistical analyses were carried out using SPSS Statistics Ver. 22 (IBM, Chicago, IL).

## Results

### Upregulation of Cyr61 was specifically induced in the CD204-positive-differentiated THP-1 cells

**Figure 1:**
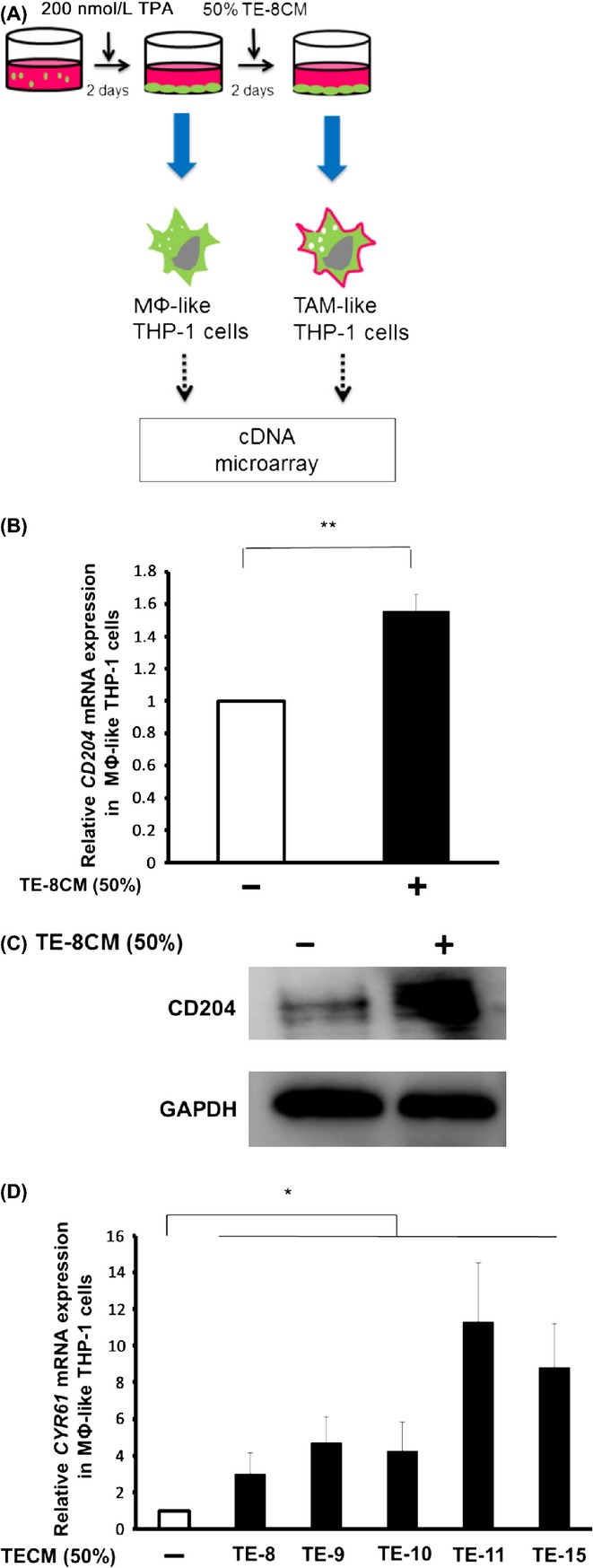
Identification of *Cyr61* differentially expressed in MΦ-like THP-1 cells treated with TECMs. (A) Schema of the strategy of cDNA microarray experiment. THP-1 cells were treated with 200 nmol/L TPA for 2 days to induce macrophage-like differentiation, and then exposed to 50% TE-8CM for 2 days. Differentially expressed genes between MΦ-like and TAM-like THP-1 cells were analyzed by cDNA microarray. (B and C) Proof of the validity of the cDNA microarray. CD204 expression was induced by TE-8CM exposure with TPA pretreatment in mRNA (B, real-time PCR) and protein (C, western blotting) levels (**P <* *0.01). (D) Confirmation of the microarray study. The expression of *CYR61* induced in TAM-like THP-1 cells was analyzed by conditioned media of esophageal cancer cell lines (*P <* *0.05). Error bars indicate SEM. TAM, tumor-associated macrophage.

First, to identify the molecules specifically upregulated in TAM-like THP-1 cells, we performed a cDNA microarray analysis using RNAs from THP-1 stimulated with 200 nmol/L TPA or with 200 nmol/L TPA followed by 50% TE-8CM (Fig.1A). To test the validity of our cDNA microarray analysis, we investigated the upregulation of CD204, a cytological marker for M2-macrophages, by TE-8CM with real-time PCR and western blotting (Fig.1B and C). Among the microarray data containing probes for 42405 human genes, 366 genes were > 2^2.5^-fold upregulated in TE-8CM-treated THP-1 cells (Table S1). We were also interested in upregulated genes known to be induced in M2 macrophages (Table2).^19^ Among these genes, we decided to focus on *CYR61*, known to promote not only macrophage polarization but also cell proliferation, invasion, survival, and metastasis of various cancers.^20^–^22^ *CYR61* was highly induced (2^2.78^-fold) by TE-8CM. The significant induction of *CYR61* mRNA by TE-8CM as well as by the other four TECMs exposure was validated in MΦ-like THP-1 cells (Fig.1D). We also observed that all of five TECMs induced Cyr61 secretion from MΦ-like THP-1 cells by enzyme-linked immunosorbent assay (ELISA) (data not shown). These results indicate that Cyr61 expression was specifically induced in the TAM-like THP-1 cells.

**Table 2:** Representative upregulated genes reported to be induced in M2 macrophages in MΦ-like THP-1 cells treated with 50% TE-8CM

Accession number	Description	Symbol	Log_2_ ratio
NM_005410	Selenoprotein P, plasma, 1, transcript variant 1	*SEPP1*	3.29
NM_024021	Membrane-spanning 4-domains, subfamily A, member 4, transcript variant 1	*MS4A4A*	2.93
NM_054034	Fibronectin 1, transcript variant 7	*FN1*	2.87
NM_001554	Cysteine-rich, angiogenic inducer, 61	*CYR61*	2.78
NM_001001547	CD36 molecule, transcript variant 2	*CD36*	2.53
NM_001557	Chemokine (C-X-C motif) receptor 2, transcript variant 1	*CXCR2*	2.59

### In the ESCC microenvironment, not only TAMs but also cancer cells expressed Cyr61

**Figure 2:**
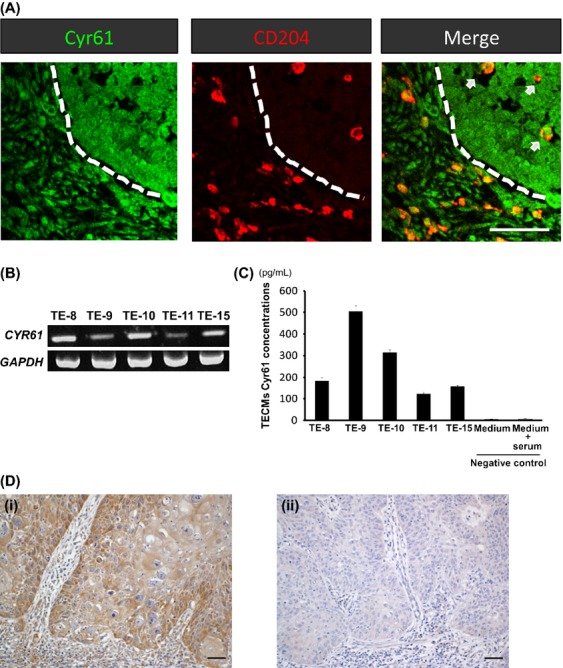
Expression of Cyr61 in the ESCC microenvironment. (A) Immunofluorescence of Cyr61 and CD204 in ESCC tissues. Cyr61 (green) was expressed in cancer nests and stroma. CD204 (red)-positive MΦ with Cyr61 expression was observed in cancer nests and stroma. Scale bar, 50 *μ*m. (B and C) Expression of Cyr61 in ESCC cell lines. Five ESCC cell lines (TE-8, TE-9, TE-10, TE-11, and TE-15) demonstrated Cyr61 mRNA expression (B, RT-PCR) and protein secretion (C, ELISA). Error bars indicate SEM. (D) Representative immunoreaction of high- and low-Cyr61 expression in the nests of ESCCs. In the high-Cyr61 group, the immunoreaction of Cyr61 was stronger than that in the corresponding nonneoplastic esophageal epithelium (i). In the low-Cyr61 group, the expression level of Cyr61 in the cancer nests was equal to that of the corresponding nonneoplastic esophageal epithelium (ii). Scale bar, 100 *μ*m. ELISA, enzyme-linked immunosorbent assay.

Cyr61 has been reported to be expressed in normal squamous epithelia and upregulated in tumor cells of squamous cell carcinoma,^23^,^24^ but its expression in stromal cells including macrophages has not been well established. We thus investigated the expression of Cyr61 in ESCC tissues by immunofluorescence (Fig.2A). The expression of Cyr61 was detected not only in cancer nests but also in stromal cells with macrophage-like morphology. Interestingly, a part of the Cyr61^+^ stromal cells also demonstrated CD204 immunoreactivity. Moreover, CD204^+^ macrophages with Cyr61 expression were evidently present within the cancer nests (Fig.2A, arrows). We also confirmed the expression of Cyr61 at the mRNA and secreted protein levels in all five ESCC cell lines (Figs.2B and C).

### The expression levels of Cyr61 showed a significant positive correlation with the number of CD204^+^ macrophages in the ESCCs

Since we observed the expression of Cyr61 in both cancer cells and macrophages, we subsequently asked whether the expression level of Cyr61 in ESCC as a whole had any statistical association with clinicopathological factors and macrophage markers. We divided the ESCC cases into high- and low-Cyr61 groups according to the relative Cyr61 staining levels of the cancer nests in comparison with that of corresponding nonneoplastic squamous epithelium (Fig.2D). The expression levels of Cyr61 in the ESCCs showed a significant positive correlation with the number of infiltrating CD204^+^ macrophages in the cancer nests and lymph node metastasis (Table3).

**Table 3:** Expression levels of Cyr61 in esophageal squamous cell carcinomas and their correlation with clinicopathological parameters and macrophage infiltration

	Number of cases	Cyr61^1^		*P* value^2^
		Low (*n *=* *45)	High (*n *=* *25)	
Histological grade^3^				
HGIEN	4	3	1	0.546
WDSCC	12	7	5	
MDSCC	43	26	17	
PDSCC	11	9	2	
Depth of tumor invasion^5^				
T1a	19	13	6	0.59
T1b	30	21	10	
T2 + T3	21	11	9	
Lymph node metastasis^5^				
Negative	43	32	11	0.026^6^
Positive	27	13	14	
Stage^4^				
0 + I	38	27	11	0.198
II + III + IV	32	18	14	
CD68^+^ cells^5^				
Low	35	23	12	0.803
High	35	22	13	
CD204^+^ cells^5^				
Low	34	26	8	0.039^6^
High	36	19	17	
CD163^+^ cells^5^				
Low	35	24	11	0.454
High	35	21	14	

ESCC, esophageal squamous cell carcinoma.

1 The relative Cyr61 staining levels of cancer nests compared to that of nonneoplastic epithelial tissues were used to divide the ESCC cases into high- and low-Cyr61 groups.

2 Data were analyzed by *χ*^2^ test.

3 According to the Japanese Classification of Esophageal Cancer.^17^ HGIEN, high-grade intraepithelial neoplasia; WDSCC, well-differentiated squamous cell carcinoma; MDSCC, moderately differentiated squamous cell carcinoma; PDSCC, poorly differentiated squamous cell carcinoma. T1a, tumor invades mucosa; T1b, tumor invades submucosa; T2, tumor invades muscularis propria; T3, tumor invades adventitia.

4 According to the TNM classification by the UICC.^18^

5 The median value of CD68^+^, CD204^+^, or CD163^+^ macrophage number of cancer nests within the areas was used to divide the patients into high and low groups.

6 *P *<* *0.05 was considered statistically significant.

### Recombinant human Cyr61 induced the expression of CD204 and promoted cell migration in MΦ-like THP-1 cells

**Figure 3:**
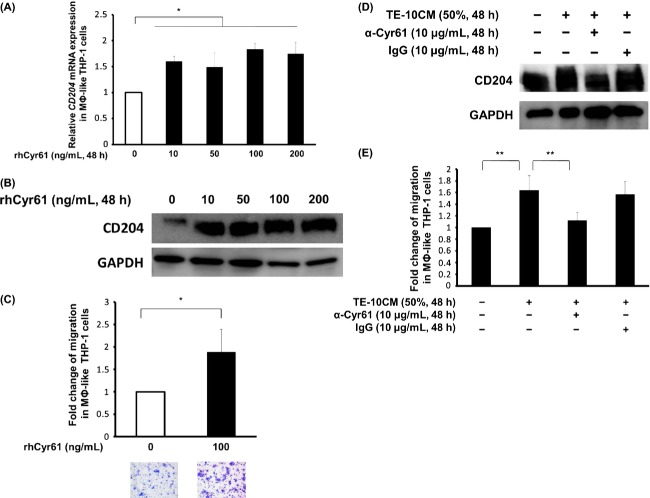
Effects of Cyr61 on the MΦ-like THP-1 cells. (A) *CD204*mRNA was significantly induced in MΦ-like THP-1 cells by rhCyr61 from 10 to 200 ng/mL (*P <* *0.05). (B) The induction of CD204 by rhCyr61 in MΦ-like THP-1 cells was confirmed by western blotting. (C) The transwell migration of MΦ-like THP-1 cells was significantly facilitated by rhCyr61 (100 ng/mL). RhCyr61 demonstrated a significant induction (1.88-fold) of migration (*P <* *0.05). (D) The expression of CD204 in MΦ-like THP-1 treated with 50% TECMs for 48 h, and pretreatment with or without neutralizing anti-Cyr61 or control IgG. Anti-Cyr61 antibody (10 *μ*g/mL) blocked the induction of CD204 by TE-10CM exposure in MΦ-like THP-1 cells. Control IgG had no effect on this phenomenon. (E) The migration of MΦ-like THP-1 by TECMs for 24 h. Anti-Cyr61 neutralizing antibody (10 *μ*g/mL) blocked the migration of MΦ-like THP-1 cells promoted by TE-10CM exposure. Control IgG had no effect on this phenomenon. (**P <* *0.01). Error bars indicate SEM.

To investigate the effect of Cyr61 on TAMs in the ESCC microenvironment, we conducted an in vitro assay using rhCyr61. Interestingly, rhCyr61 (10–200 ng/mL) induced the expression of CD204 in MΦ-like THP-1 cells at mRNA and protein levels (Fig.3A and B). Moreover, in the transwell assay, rhCyr61 (100 ng/mL) facilitated the migration of MΦ-like THP-1 cells (Fig.3C). We then verified the role of Cyr61 from cancer cells in the promotion of CD204 and promotion of MΦ migration in the ESCC microenvironment. Not only the significant induction of CD204 by TE-10CM exposure (Fig.3D) but also the TECM-induced migration of MΦ-like THP-1 cells was blocked by the addition of anti-Cyr61 antibody into the culture media (Fig.3E).

### MEK/ERK pathway was involved in the Cyr61-mediated migration and CD204 expression in MΦ-like THP-1 cells

**Figure 4:**
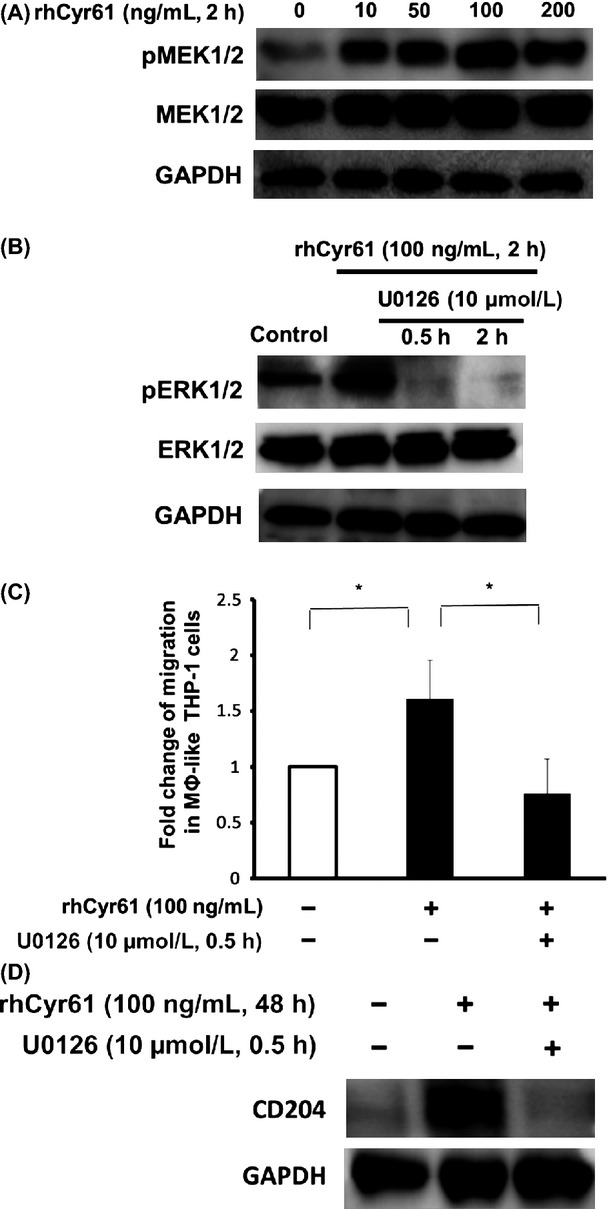
The involvement of MEK/ERK pathway in the effect of rhCyr61 on THP-1 cells. (A) MΦ-like THP-1 cells were treated with rhCyr61 at the indicated dosages for 2 h. An induction of the phosphorylation of MEK1/2 by rhCyr61 was detected. (B) MΦ-like THP-1 cells were pretreated with the MEK1/2 inhibitor U0126 (10 *μ*mol/L) for 0.5 or 2 h, then stimulated with rhCyr61 (100 ng/mL) for 2 h. The phosphorylation of Erk1/2, a downstream signaling molecule, was inhibited. (C) The transwell migration of MΦ-like THP-1 cells promoted by rhCyr61 was significantly inhibited by U0126 pretreatment. U0126 demonstrated significant inhibition of rhCyr61-induced migration (*P <* *0.05). Error bars indicate SEM. (D) U0126 pretreatment significantly inhibited the induction of CD204 expression in MΦ-like THP-1 cells by rhCyr61.

As MEK/ERK pathways are known to be involved in the Cyr61-mediated recruitment of macrophages,^25^ we hypothesized that the MEK/ERK pathway may be activated by Cyr61 in MΦ-like THP-1 cells. The MEK1/2 phosphorylation was increased in response to rhCyr61 stimulation (10–200 ng/mL) (Fig.4A). Pretreatment with a MEK1/2 inhibitor U0126 at 10 *μ*mol/L for 0.5 or 2 h significantly inhibited both ERK1/2 phosphorylation and the Cyr61-mediated migration (Fig.4B and C). Interestingly, CD204 expression was also inhibited by U0126 treatment at 10 *μ*mol/L for 0.5 h (Fig.4D).

## Discussion

In this study, to investigate the role of TAMs in ESCC, we extracted the upregulated genes reported as M2 genes in MΦ-like THP-1 cells by conditioned medium of ESCC cell line with a cDNA microarray analysis as described.^19^ Among the genes, *CYR61* was induced in TAM-like THP-1 cells. Cyr61 (also called CCN1) is a component of the extracellular matrix which belongs to the CCN protein family.^26^–^28^ Cyr61 is considered to be an angiogenesis inducer.^26^ *CYR61*-null mice suffer developmental failure and embryonic death due to vascular defects in the placenta.^29^ Cyr61 was reported to express on the vessel wall of the developmental circulatory system and regulates the proliferation of vessel endothelial cells and angiogenesis.^30^ Cyr61 is composed of four conserved modular domains.31With these specific modular domains, Cyr61 has a wide variety of biological functions.^26^

In terms of cancer, the overexpression of Cyr61 has been linked to the growth and progression of various cancers.^27^ Cyr61 has also been reported to support the chemotaxis of various cell types including murine macrophages,^25^,^32^ and to induce a proinflammatory genetic program of murine macrophages.^32^ From these reports, we speculate that Cyr61 might also be involved in the recruitment or differentiation of macrophages in the ESCC microenvironment.

In this study, Cyr61 expression and secretion were significantly induced by TECMs in MΦ-like THP-1 cells. This phenomenon indicated that humoral factor(s) from the cancer cells might be responsible for the Cyr61 induction in macrophages. In our immunofluorescence examination, Cyr61 was detected in cancer cells as well as stromal cells of ESCC tissues. Moreover, part of the Cyr61-positive stromal cells expressed CD204 simultaneously. These findings suggested that not only cancer cells but also TAMs expressed Cyr61 in the ESCC microenvironment. To our knowledge, there are only a few reports describing Cyr61 expression in the cancer stroma of ESCC.^33^ Moreover, Cyr61 transcript was reported to be more abundantly expressed in invasive TAMs than general TAMs in a mouse model.^34^ In addition, Stewart et al. defined *Cyr61* as one of the M2 genes.^19^ These reports support our findings of Cyr61 expression in ESCC tissues. Our present findings may provide new knowledge with regard to Cyr61 expression in the ESCC microenvironment.

The overexpression of Cyr61 in ESCC tissues has been reported to be associated with poor survival of ESCC patients, although there was no association between Cyr61 expression and clinicopathological variables including histological grade, clinical stage, and lymph node metastasis.^33^,^35^ We also confirmed that the overall expression levels of Cyr61 showed a significant positive correlation with only lymph node metastasis in our series of ESCC cases. We also assessed the correlation between the expression levels of Cyr61 and macrophage infiltration for the first time. Interestingly, the number of infiltrating CD204^+^ macrophages but not those of other macrophage markers including CD68 and CD163 showed a significant positive correlation with the overall expression levels of Cyr61 in ESCC. As we already reported that CD204 was a useful marker for TAMs contributing to the angiogenesis, progression, and prognosis of ESCC,^10^ We speculated that Cyr61 might associated with the aggressiveness of ESCC by the induction of cell migration and CD204 expression in macrophages.

In accordance with previous reports,^25^,^32^ we observed here that rhCyr61 protein promoted the migration of MΦ-like THP-1 cells. We found that the expression of CD204, an M2-marker, was also induced by rhCyr61. In contrast, other M2 factors, including *CD163*, *IL-10*, *TGF-β*, *VEGFA*, *VEGFC*, *MMP2*, and *MMP9* were tended to be upregulated. Conversely, M1 factors, including *IL-6*, *IFN-γ*, and *TNF-α* showed tendency to be downregulated by rhCyr61 not reaching the statistical significance (data not shown). Moreover, we observed that a neutralizing anti-Cyr61 antibody blocked not only the migration but also the CD204 expression in MΦ-like THP-1 cells promoted by TECM exposure. The effect of Cyr61 on CD204 expression in macrophages has not yet been reported. Mantovani et al. suggested that IL-10 might be a factor inducing CD204 expression.^2^ Among the five ESCC cell lines used in this study, only TE-10 demonstrated a significant amount of IL-10 secretion (data not shown). Cyr61 might thus be one of the important inducers of CD204 in macrophages.

Cyr61 has been shown to recruit macrophages through the activation of the MEK/ERK signaling pathway in myeloid-derived macrophage and mouse macrophage cell lines in vitro.^25^ Indeed, we detected the activation of the MEK/ERK pathway in MΦ-like THP-1 cells by rhCyr61 treatment. In addition, pretreatment with U0126, a MEK1/2 inhibitor, significantly inhibited not only the Cyr61-mediated migration but also the CD204 expression. NF-*κ*B, Stat3, Stat6, c-myc, and interferon regulatory factor are involved in skewing to the M2 phenotype of macrophages.^36^–^39^ Among them, it is well known that Stat3 is an important transcription factor in the interaction between TAMs and tumor cells.^40^–^42^ However, the distinct pathway of CD204 expression in macrophages is not completely understood. There are some reports investigated the function of CD204. Ohnishi et al. suggested that CD204 suppresses the MΦ activation by inhibiting the binding of LPS to toll-like receptor 4 and regulates the inflammatory cytokine production.^43^ Neyen et al. showed that CD204 on MΦ is important for tumor progression and metastasis in vitro and in vivo.^44^ Our findings may suggest a new mechanism for the regulation of CD204 expression.

In conclusion, it cannot be overlooked that not only cancer cells but also TAMs express Cyr61 in the ESCC microenvironment. In addition, the role of Cyr61 for macrophages in the ESCC microenvironment should be taken into consideration. In this study, our overall results overall indicate that Cyr61 contributes to the expression of CD204 and the promotion of cell migration *via* MEK/ERK pathway in TAMs of ESCCs within their specific tumor microenvironment.

## Supporting Information

**Table S1.** The genes differentially upregulated (>2^2.5^-fold) in MΦ-like THP-1 cells treated with 50% TE-8CM.
