# Supplementary material for: Cyr61 promotes CD204 expression and the migration of macrophages via MEK/ERK pathway in esophageal squamous cell carcinoma
Source: Cancer Med. 2015 Jan 26;4(3):437–46. doi: 10.1002/cam4.401 (PMC4380969; doi:10.1002/cam4.401)
Supplement: Supplementary file 1 [file cam40004-0437-sd1.docx]

Supplementary Table S1. The genes differentially upregulated (> 2^2.5^-fold) in MΦ-like THP-1 cells treated with 50% TE-8CM

| Accession number | Description | Symbol | Log2 ratio |
| --- | --- | --- | --- |
| NM_001067 | topoisomerase (DNA) II alpha 170kDa | *TOP2A* | 7.16 |
| NM_004878 | prostaglandin E synthase | *PTGES* | 6.93 |
| NM_018685 | anillin, actin binding protein | *ANLN* | 6.75 |
| NM_202002 | forkhead box M1, transcript variant 1 | *FOXM1* | 6.53 |
| NM_001237 | cyclin A2 | *CCNA2* | 6.36 |
| NM_005030 | polo-like kinase 1 | *PLK1* | 6.34 |
| NM_005733 | kinesin family member 20A | *KIF20A* | 6.31 |
| NM_014750 | discs, large (Drosophila) homolog-associated protein 5, transcript variant 1 | *DLGAP5* | 6.29 |
| NM_001790 | cell division cycle 25 homolog C (S. pombe), transcript variant 1 | *CDC25C* | 6.21 |
| NM_018136 | asp (abnormal spindle) homolog, microcephaly associated (Drosophila), transcript variant 1 | *ASPM* | 6.06 |
| NM_000491 | complement component 1, q subcomponent, B chain | *C1QB* | 6.00 |
| NM_012310 | kinesin family member 4A | *KIF4A* | 6.00 |
| NM_012484 | ns hyaluronan-mediated motility receptor (HMMR), transcript variant 2 | *HMMR* | 5.98 |
| NM_000948 | prolactin (PRL), transcript variant 1 | *PRL* | 5.95 |
| NM_001039535 | spindle and kinetochore associated complex subunit 1, transcript variant 1 | *SKA1* | 5.89 |
| NM_003318 | TTK protein kinase, transcript variant 1 | *TTK* | 5.71 |
| NM_018131 | centrosomal protein 55kDa, transcript variant 1 | *CEP55* | 5.70 |
| NM_005192 | cyclin-dependent kinase inhibitor 3, transcript variant 1 | *CDKN3* | 5.63 |
| NM_152562 | cell division cycle associated 2 | *CDCA2* | 5.56 |
| NM_002497 | NIMA (never in mitosis gene a)-related kinase 2, transcript variant 1 | *NEK2* | 5.55 |
| NM_001080416 | v-myb myeloblastosis viral oncogene homolog (avian)-like 1, transcript variant 1 | *MYBL1* | 5.53 |
| NM_024094 | defective in sister chromatid cohesion 1 homolog | *DSCC1* | 5.52 |
| NM_001786 | cyclin-dependent kinase 1, transcript variant 1 | *CDK1* | 5.52 |
| NM_003686 | exonuclease 1 EXO1, transcript variant 3 | *EXO1* | 5.49 |
| NM_016426 | G-2 and S-phase expressed 1 | *GTSE1* | 5.46 |
| NM_001211 | budding uninhibited by benzimidazoles 1 homolog beta (yeast) | *BUB1B* | 5.44 |
| NM_005814 | glycoprotein A33 (transmembrane) | *GPA33* | 5.41 |
| NM_145061 | spindle and kinetochore associated complex subunit 3 | *SKA3* | 5.38 |
| NM_004091 | E2F transcription factor 2 | *E2F2* | 5.37 |
| NM_006845 | kinesin family member 2C | *KIF2C* | 5.36 |
| NM_024680 | E2F transcription factor 8 | *E2F8* | 5.33 |
| NM_007174 | citron (rho-interacting, serine/threonine kinase 21), transcript variant 2 | *CIT* | 5.31 |
| NM_018410 | Holliday junction recognition protein | *HJURP* | 5.29 |
| NM_018369 | DEP domain containing 1B, transcript variant 1 | *DEPDC1B* | 5.29 |
| NM_001012271 | baculoviral IAP repeat containing 5, transcript variant 3 | *BIRC5* | 5.28 |
| NM_001004343 | microtubule-associated protein 1 light chain 3 gamma | *MAP1LC3C* | 5.26 |
| NM_016359 | nucleolar and spindle associated protein 1, transcript variant 1 | *NUSAP1* | 5.26 |
| NM_172369 | complement component 1, q subcomponent, C chain, transcript variant 2 | *C1QC* | 5.26 |
| NM_002417 | antigen identified by monoclonal antibody Ki-67, transcript variant 1 | *MKI67* | 5.26 |
| NM_006101 | NDC80 homolog, kinetochore complex component (S. cerevisiae) | *NDC80* | 5.20 |
| NM_022346 | non-SMC condensin I complex, subunit G | *NCAPG* | 5.19 |
| NM_000569 | Fc fragment of IgG, low affinity IIIa, receptor (CD16a), transcript variant 1 | *FCGR3A* | 5.19 |
| NM_018492 | PDZ binding kinase | *PBK* | 5.18 |
| NM_012112 | TPX2, microtubule-associated, homolog (Xenopus laevis) | *TPX2* | 5.17 |
| NM_001809 | centromere protein A, transcript variant 1 | *CENPA* | 5.13 |
| NM_080668 | cell division cycle associated 5 | *CDCA5* | 5.12 |
| NM_199420 | polymerase (DNA directed), theta | *POLQ* | 5.12 |
| NM_020242 | kinesin family member 15 | *KIF15* | 5.10 |
| NM_007280 | Opa interacting protein 5 | *OIP5* | 5.09 |
| NM_002029 | formyl peptide receptor 1, transcript variant 2 | *FPR1* | 5.09 |
| NM_001002876 | centromere protein M, transcript variant 2, | *CENPM* | 5.07 |
| NM_145697 | NUF2, NDC80 kinetochore complex component, homolog (S. cerevisiae), transcript variant 1 | *NUF2* | 5.06 |
| NM_002612 | pyruvate dehydrogenase kinase, isozyme 4, nuclear gene encoding mitochondrial protein | *PDK4* | 5.02 |
| NM_031217 | kinesin family member 18A | *KIF18A* | 5.02 |
| NM_017779 | DEP domain containing 1, transcript variant 2 | *DEPDC1* | 5.02 |
| NM_001255 | cell division cycle 20 homolog (S. cerevisiae) | *CDC20* | 5.00 |
| NM_015653 | RIB43A domain with coiled-coils 2 | *RIBC2* | 4.98 |
| NM_002964 | S100 calcium binding protein A8 | *S100A8* | 4.97 |
| NM_003579 | RAD54-like (S. cerevisiae), transcript variant 1 | *RAD54L* | 4.96 |
| NM_016448 | denticleless homolog (Drosophila) | *DTL* | 4.96 |
| NM_002263 | kinesin family member C1 | *KIFC1* | 4.92 |
| NM_003981 | protein regulator of cytokinesis 1, transcript variant 1 | *PRC1* | 4.91 |
| NM_001048166 | SCL/TAL1 interrupting locus, transcript variant 1, | *STIL* | 4.87 |
| NM_014875 | kinesin family member 14 | *KIF14* | 4.87 |
| NM_001442 | fatty acid binding protein 4, adipocyte | *FABP4* | 4.86 |
| NM_001005464 | histone cluster 2, H3a, | *HIST2H3A* | 4.85 |
| NM_001462 | formyl peptide receptor 2, transcript variant 1 | *FPR2* | 4.85 |
| NM_014791 | maternal embryonic leucine zipper kinase MELK | *MELK* | 4.84 |
| NM_001017420 | establishment of cohesion 1 homolog 2 (S. cerevisiae) | *ESCO2* | 4.82 |
| NM_003509 | histone cluster 1, H2ai | *HIST1H2AI* | 4.82 |
| NM_022111 | claspin, transcript variant 1, | *CLSPN* | 4.82 |
| NM_020675 | SPC25, NDC80 kinetochore complex component, homolog (S. cerevisiae) | *SPC25* | 4.81 |
| NM_001042517 | aphanous homolog 3 (Drosophila), transcript variant 1 | *DIAPH3* | 4.77 |
| NM_015687 | filamin A interacting protein 1 | *FILIP1* | 4.74 |
| NM_001034 | ribonucleotide reductase M2 | *RRM2* | 4.71 |
| NM_004336 | budding uninhibited by benzimidazoles 1 homolog (yeast) | *BUB1* | 4.70 |
| NM_003258 | thymidine kinase 1, soluble | *TK1* | 4.69 |
| NM_001071 | thymidylate synthetase | *TYMS* | 4.67 |
| NM_130386 | collectin sub-family member 12 | *COLEC12* | 4.66 |
| NM_012415 | RAD54 homolog B (S. cerevisiae) | *RAD54B* | 4.63 |
| NM_030919 | family with sequence similarity 83, member D | *FAM83D* | 4.62 |
| NM_014353 | RAB26, member RAS oncogene family | *RAB26* | 4.60 |
| NM_018101 | cell division cycle associated 8 | *CDCA8* | 4.59 |
| NM_004523 | kinesin family member 11 | *KIF11* | 4.59 |
| NM_203401 | stathmin 1, transcript variant 1 | *STMN1* | 4.58 |
| NM_006479 | RAD51 associated protein 1, transcript variant 2, | *RAD51AP1* | 4.57 |
| NM_002692 | polymerase (DNA directed), epsilon 2 (p59 subunit), transcript variant 1, | *POLE2* | 4.56 |
| NM_001195228 | family with sequence similarity 64, member A | *FAM64A* | 4.55 |
| NM_016343 | centromere protein F, 350/400kDa (mitosin) | *CENPF* | 4.54 |
| NM_052960 | retinol binding protein 7, cellular (RBP7) | *RBP7* | 4.52 |
| NM_031966 | cyclin B1 | *CCNB1* | 4.51 |
| NM_148674 | tructural maintenance of chromosomes 1B | *SMC1B* | 4.50 |
| NM_014264 | polo-like kinase 4, transcript variant 1, | *PLK4* | 4.50 |
| NM_018154 | ASF1 anti-silencing function 1 homolog B (S. cerevisiae) | *ASF1B* | 4.47 |
| NM_199357 | Rho GTPase activating protein 11A | *ARHGAP11A* | 4.47 |
| NM_001012409 | shugoshin-like 1 (S. pombe), transcript variant A1 | *SGOL1* | 4.45 |
| NM_194277 | FERM domain containing 7 | *FRMD7* | 4.45 |
| NM_018248 | nei endonuclease VIII-like 3 (E. coli) | *NEIL3* | 4.44 |
| NM_001142651 | neuralized homolog 1B (Drosophila) | *NEURL1B* | 4.44 |
| NM_020163 | sema domain, immunoglobulin domain (Ig), short basic domain, secreted, (semaphorin) 3G | *SEMA3G* | 4.42 |
| NM_181803 | ubiquitin-conjugating enzyme E2C, transcript variant 6, | *UBE2C* | 4.41 |
| NM_181558 | eplication factor C (activator 1) 3, 38kDa, transcript variant 2, | *RFC3* | 4.40 |
| NM_138555 | kinesin family member 23, transcript variant 1 | *KIF23* | 4.40 |
| NM_001813 | centromere protein E, 312kDa | *CENPE* | 4.40 |
| NM_005225 | E2F transcription factor 1 | *E2F1* | 4.38 |
| NM_032997 | ZW10 interactor, transcript variant 2, | *ZWINT* | 4.37 |
| NM_004217 | aurora kinase B | *AURKB* | 4.36 |
| NM_002466 | v-myb myeloblastosis viral oncogene homolog (avian)-like 2 | *MYBL2* | 4.24 |
| NM_015430 | peptidase domain containing associated with muscle regeneration 1, transcript variant 1 | *PAMR1* | 4.22 |
| NM_152515 | cytoskeleton associated protein 2-like | *CKAP2L* | 4.21 |
| NM_003504 | cell division cycle 45 homolog (S. cerevisiae), transcript variant 2, | *CDC45* | 4.19 |
| NM_016095 | GINS complex subunit 2 (Psf2 homolog) | *GINS2* | 4.18 |
| NM_002358 | MAD2 mitotic arrest deficient-like 1 (yeast) | *MAD2L1* | 4.17 |
| NM_182687 | protein kinase, membrane associated tyrosine/threonine 1, transcript variant 2 | *PKMYT1* | 4.16 |
| NM_152308 | RMI2, RecQ mediated genome instability 2, homolog (S. cerevisiae) | *RMI2* | 4.15 |
| NM_007286 | synaptopodin, transcript variant 1 | SYNPO | 4.14 |
| NR_002947 | esticular cell adhesion molecule 1 homolog (mouse), pseudogene, non-coding RNA | *TCAM1P* | 4.06 |
| NM_024857 | ATPase family, AAA domain containing 5 | *ATAD5* | 4.06 |
| NM_014321 | origin recognition complex, subunit 6, transcript variant 1 | *ORC6* | 4.04 |
| NM_013277 | Rac GTPase activating protein 1, transcript variant 1 | *RACGAP1* | 4.04 |
| NM_018193 | Fanconi anemia, complementation group I, transcript variant 2 | *FANCI* | 4.03 |
| NM_024745 | SHC SH2-domain binding protein 1 | *SHCBP1* | 4.03 |
| NM_004153 | origin recognition complex, subunit 1, transcript variant 1 | *ORC1* | 4.02 |
| NM_003878 | gamma-glutamyl hydrolase (conjugase, folylpolygammaglutamyl hydrolase) | *GGH* | 4.01 |
| NM_138419 | family with sequence similarity 54, member A, transcript variant 2 | *FAM54A* | 4.00 |
| NM_182751 | inichromosome maintenance complex component 10, transcript variant 1 | *MCM10* | 3.99 |
| NM_020394 | zinc finger protein 695, transcript variant 1 | *ZNF695* | 3.99 |
| NM_025049 | PIF1 5'-to-3' DNA helicase homolog (S. cerevisiae) | *PIF1* | 3.96 |
| NM_001161616 | ral guanine nucleotide dissociation stimulator-like 3, transcript variant 1 | *RGL3* | 3.94 |
| NM_031942 | cell division cycle associated 7, transcript variant 1 | *CDCA7* | 3.92 |
| NM_004795 | klotho | *KL* | 3.91 |
| NM_003986 | butyrobetaine (gamma), 2-oxoglutarate dioxygenase (gamma-butyrobetaine hydroxylase) 1 | *BBOX1* | 3.91 |
| NM_002404 | microfibrillar-associated protein 4, transcript variant 2 | *MFAP4* | 3.90 |
| NM_030928 | chromatin licensing and DNA replication factor 1 | *CDT1* | 3.88 |
| NM_004293 | guanine deaminase, transcript variant 2 | *GDA* | 3.86 |
| NM_002315 | LIM domain only 1 (rhombotin 1) | *LMO1* | 3.86 |
| NM_002875 | RAD51 homolog (S. cerevisiae), transcript variant 1 | *RAD51* | 3.86 |
| NM_170589 | cancer susceptibility candidate 5, transcript variant 1, | *CASC5* | 3.84 |
| NM_002899 | retinol binding protein 1, cellular, transcript variant 1 | *RBP1* | 3.83 |
| NM_000798 | dopamine receptor D5 | *DRD5* | 3.83 |
| NM_031965 | germ cell associated 2 (haspin) | *GSG2* | 3.82 |
| NM_004265 | fatty acid desaturase 2 | *FADS2* | 3.81 |
| NM_198947 | family with sequence similarity 111, member B, transcript variant 1 | *FAM111B* | 3.81 |
| NM_014109 | ATPase family, AAA domain containing 2 | *ATAD2* | 3.76 |
| NM_018063 | helicase, lymphoid-specific | *HELLS* | 3.75 |
| NM_003511 | histone cluster 1, H2al | *HIST1H2AL* | 3.73 |
| NM_022785 | EF-hand calcium binding domain 6 | *EFCAB6* | 3.73 |
| NM_000838 | glutamate receptor, metabotropic 1 | *GRM1* | 3.72 |
| NM_002965 | S100 calcium binding protein A9 | *S100A9* | 3.72 |
| NM_053034 | anthrax toxin receptor 1, transcript variant 2 | *ANTXR1* | 3.69 |
| NM_004169 | serine hydroxymethyltransferase 1 (soluble), nuclear gene encoding mitochondrial protein, transcript variant 1 | *SHMT1* | 3.69 |
| NM_024908 | WD repeat domain 76, transcript variant 1 | *WDR76* | 3.69 |
| NM_001018115 | Fanconi anemia, complementation group D2, transcript variant 2 | *FANCD2* | 3.67 |
| NM_152487 | transmembrane protein 56, transcript variant 2 | *TMEM56* | 3.67 |
| NM_024734 | calmin (calponin-like, transmembrane) | *CLMN* | 3.67 |
| NM_021067 | GINS complex subunit 1 (Psf1 homolog) | *GINS1* | 3.67 |
| NM_001159 | aldehyde oxidase 1 | *AOX1* | 3.67 |
| NM_001789 | cell division cycle 25 homolog A (S. pombe), transcript variant 1, | *CDC25A* | 3.66 |
| NM_201649 | solute carrier family 6 (neurotransmitter transporter, glycine), member 9, transcript variant 2 | *SLC6A9* | 3.64 |
| NM_152358 | IZUMO family member 2 | *IZUMO2* | 3.64 |
| NM_007109 | ranscription factor 19, transcript variant 1, | *TCF19* | 3.64 |
| NM_005441 | chromatin assembly factor 1, subunit B (p60) | *CHAF1B* | 3.63 |
| NM_018304 | proline rich 11 | *PRR11* | 3.61 |
| NM_032571 | egf-like module containing, mucin-like, hormone receptor-like 3 | *EMR3* | 3.61 |
| NM_001845 | collagen, type IV, alpha 1 | *COL4A1* | 3.59 |
| NM_022145 | centromere protein K | *CENPK* | 3.59 |
| NM_020200 | hosphoribosyl transferase domain containing 1 | *PRTFDC1* | 3.58 |
| NM_001613 | actin, alpha 2, smooth muscle, aorta, transcript variant 2 | *ACTA2* | 3.56 |
| NM_201553 | ibrinogen-like 1, transcript variant 4 | *FGL1* | 3.56 |
| NM_001005785 | eleted in azoospermia 2, transcript variant 2 | *DAZ2* | 3.55 |
| NM_001122962 | signal-regulatory protein beta 2, transcript variant 1 | *SIRPB2* | 3.55 |
| NM_031299 | cell division cycle associated 3 | *CDCA3* | 3.54 |
| NM_003004 | secreted and transmembrane 1 | *SECTM1* | 3.53 |
| NM_005423 | trefoil factor 2 | *TFF2* | 3.52 |
| NM_080671 | potassium voltage-gated channel, Isk-related family, member 4 | *KCNE4* | 3.52 |
| NM_178233 | otopetrin 3 | *OTOP3* | 3.51 |
| NM_183240 | transmembrane protein 37 | *TMEM37* | 3.51 |
| NM_000057 | Bloom syndrome, RecQ helicase-like | *BLM* | 3.50 |
| NM_018555 | zinc finger protein 331, transcript variant 1 | *ZNF331* | 3.50 |
| NM_004119 | fms-related tyrosine kinase 3 | *FLT3* | 3.50 |
| NM_013290 | PSMC3 interacting protein, transcript variant 1 | *PSMC3IP* | 3.50 |
| NM_004701 | cyclin B2 | *CCNB2* | 3.50 |
| NM_005980 | S100 calcium binding protein P | *S100P* | 3.50 |
| NM_032152 | PML-RARA regulated adaptor molecule 1 | *PRAM1* | 3.49 |
| NM_005322 | histone cluster 1, H1b | *HIST1H1B* | 3.48 |
| NM_024629 | MLF1 interacting protein | *MLF1IP* | 3.48 |
| NM_024629 | integrin-binding sialoprotein | *IBSP* | 3.48 |
| NM_030771 | coiled-coil domain containing 34 | *CCDC34* | 3.46 |
| NM_007300 | breast cancer 1, early onset, transcript variant 2 | *BRCA1* | 3.44 |
| NM_001005179 | olfactory receptor, family 56, subfamily A, member 4 | *OR56A4* | 3.44 |
| NM_006733 | centromere protein I | *CENPI* | 3.42 |
| NM_198468 | MMS22-like, DNA repair protein | *MMS22L* | 3.42 |
| NM_031476 | cysteine-rich secretory protein LCCL domain containing 2 | *CRISPLD2* | 3.42 |
| NM_014708 | kinetochore associated 1 | *KNTC1* | 3.39 |
| NM_032336 | INS complex subunit 4 (Sld5 homolog) | *GINS4* | 3.38 |
| NM_003537 | histone cluster 1, H3b | *HIST1H3B* | 3.37 |
| NM_007331 | Wolf-Hirschhorn syndrome candidate 1, transcript variant 8 | *WHSC1* | 3.36 |
| NM_004307 | amyloid beta (A4) precursor protein-binding, family B, member 2, transcript variant 1 | *APBB2* | 3.36 |
| NM_001001918 | olfactory receptor, family 14, subfamily C, member 36 | *OR14C36* | 3.35 |
| NM_007317 | kinesin family member 22 | *KIF22* | 3.34 |
| NM_005480 | trophinin associated protein (tastin), transcript variant 1 | *TROAP* | 3.34 |
| NM_032043 | BRCA1 interacting protein C-terminal helicase 1 | *BRIP1* | 3.34 |
| NM_001185156 | interleukin 24, transcript variant 3 | *IL24* | 3.34 |
| NM_138286 | zinc finger protein 681 | *ZNF681* | 3.34 |
| NM_001254 | cell division cycle 6 homolog | *CDC6* | 3.33 |
| NM_001145513 | secernin 1, transcript variant 1 | *SCRN1* | 3.32 |
| NM_000567 | C-reactive protein, pentraxin-related | *CRP* | 3.31 |
| NM_057749 | cyclin E2 | *CCNE2* | 3.30 |
| NM_006342 | transforming, acidic coiled-coil containing protein 3 | *TACC3* | 3.30 |
| NM_002253 | kinase insert domain receptor (a type III receptor tyrosine kinase) | *KDR* | 3.30 |
| NM_005410 | selenoprotein P, plasma, 1, transcript variant 1 | *SEPP1* | 3.29 |
| NM_005282 | G protein-coupled receptor 4 | *GPR4* | 3.29 |
| NM_013402 | fatty acid desaturase 1 | *FADS1* | 3.28 |
| NM_001039841 | Rho GTPase activating protein 11B | *ARHGAP11B* | 3.27 |
| NM_006486 | fibulin 1 transcript variant D, | *FBLN1* | 3.27 |
| NM_001826 | CDC28 protein kinase regulatory subunit 1B, transcript variant 1, | *CKS1B* | 3.27 |
| NM_002302 | leukocyte cell-derived chemotaxin 2 | *LECT2* | 3.26 |
| NM_145287 | zinc finger protein 519, transcript variant 1 | *ZNF519* | 3.26 |
| NM_020405 | plexin domain containing 1 | *PLXDC1* | 3.25 |
| NM_001138 | agouti related protein homolog (mouse) | *AGRP* | 3.21 |
| NM_006461 | sperm associated antigen 5 | *SPAG5* | 3.20 |
| NM_021992 | thymosin beta 15a | *TMSB15A* | 3.19 |
| NM_000946 | primase, DNA, polypeptide 1 (49kDa) | *PRIM1* | 3.19 |
| NM_006033 | lipase, endothelial | *LIPG* | 3.17 |
| NM_005601 | natural killer cell group 7 sequence | *NKG7* | 3.17 |
| NM_018945 | phosphodiesterase 7B | *PDE7B* | 3.17 |
| NM_001018112 | Fanconi anemia, complementation group A, transcript variant 2 | *FANCA* | 3.15 |
| NM_005879 | TRAF interacting protein | *TRAIP* | 3.15 |
| NM_004260 | RecQ protein-like 4 | *RECQL4* | 3.15 |
| NM_015991 | complement component 1, q subcomponent, A chain | *C1QA* | 3.14 |
| NM_000493 | collagen, type X, alpha 1 | *COL10A1* | 3.13 |
| NM_004900 | apolipoprotein B mRNA editing enzyme, catalytic polypeptide-like 3B | *APOBEC3B* | 3.13 |
| NM_001047160 | neuroepithelial cell transforming 1, transcript variant 1 | *NET1* | 3.13 |
| NM_001193552 | zinc finger protein 850 | *ZNF850* | 3.13 |
| NM_014419 | dickkopf-like 1, transcript variant 1 | *DKKL1* | 3.13 |
| NM_001029875 | regulator of G-protein signaling 7 binding protein | *RGS7BP* | 3.11 |
| NM_001174104 | CD14 molecule, transcript variant 3 | *CD14* | 3.11 |
| NM_020980 | aquaporin 9 | *AQP9* | 3.11 |
| NM_016195 | kinesin family member 20B | *KIF20B* | 3.10 |
| NM_004086 | coagulation factor C homolog, cochlin (Limulus polyphemus), transcript variant 2, | *COCH* | 3.10 |
| NM_194313 | kinesin family member 24 | *KIF24* | 3.08 |
| NM_000331 | serum amyloid A1, transcript variant 1 | *SAA1* | 3.08 |
| NM_001351 | deleted in azoospermia-like, transcript variant 2 | *DAZL* | 3.08 |
| NM_001039 | odium channel, nonvoltage-gated 1, gamma | *SCNN1G* | 3.08 |
| NM_001083961 | WD repeat domain 62, transcript variant 1 | *WDR62* | 3.07 |
| NM_153819 | RAS guanyl releasing protein 2 (calcium and DAG-regulated), transcript variant 2, | *RASGRP2* | 3.06 |
| NM_001408 | cadherin, EGF LAG seven-pass G-type receptor 2 (flamingo homolog, Drosophila) | *CELSR2* | 3.06 |
| NM_005143 | haptoglobin, transcript variant 1, | *HP* | 3.05 |
| NM_000165 | gap junction protein, alpha 1, 43kDa | *GJA1* | 3.05 |
| NM_018365 | meiosis-specific nuclear structural 1 | *MNS1* | 3.05 |
| NM_025092 | ATH1, acid trehalase-like 1 (yeast) | *ATHL1* | 3.05 |
| NM_001775 | CD38 molecule | *CD38* | 3.05 |
| NM_017669 | excision repair cross-complementing rodent repair deficiency, complementation group 6-like | *ERCC6L* | 3.04 |
| NM_080860 | radial spoke head 1 homolog (Chlamydomonas) | *RSPH1* | 3.04 |
| NM_022843 | protocadherin 20 | *PCDH20* | 3.03 |
| NM_006288 | Thy-1 cell surface antigen | *THY1* | 3.03 |
| NM_003178 | synapsin II, transcript variant IIb | *SYN2* | 3.03 |
| NM_002585 | pre-B-cell leukemia homeobox 1, transcript variant 1 | *PBX1* | 3.02 |
| NM_001037954 | DIX domain containing 1, transcript variant 1 | *DIXDC1* | 3.02 |
| NM_006056 | neuromedin U receptor 1 | *NMUR1* | 3.01 |
| NM_007086 | WD repeat and HMG-box DNA binding protein 1, transcript variant 1 | *WDHD1* | 3.00 |
| NM_153695 | zinc finger protein 367 | *ZNF367* | 3.00 |
| NM_013244 | mannosyl (alpha-1,3-)-glycoprotein beta-1,4-N-acetylglucosaminyltransferase, isozyme C (putative) | *MGAT4C* | 2.99 |
| NM_002116 | major histocompatibility complex, class I, A, transcript variant 1 | *HLA-A* | 2.99 |
| NM_007068 | DMC1 dosage suppressor of mck1 homolog, meiosis-specific homologous recombination (yeast) | *DMC1* | 2.99 |
| NM_182734 | phospholipase C, beta 1 (phosphoinositide-specific), transcript variant 2, | *PLCB1* | 2.98 |
| NM_014228 | solute carrier family 6 (neurotransmitter transporter, L-proline), member 7 | *SLC6A7* | 2.98 |
| NM_001001557 | growth differentiation factor 6 | *GDF6* | 2.97 |
| NM_032607 | cAMP responsive element binding protein 3-like 3 | *CREB3L3* | 2.96 |
| NM_000059 | breast cancer 2, early onset | *BRCA2* | 2.95 |
| NM_001031853 | inscuteable homolog (Drosophila), transcript variant 1 | *INSC* | 2.95 |
| NM_152380 | T-box 15 | *TBX15* | 2.95 |
| NM_031303 | katanin p60 subunit A-like 2 | *KATNAL2* | 2.94 |
| NM_017923 | membrane-associated ring finger (C3HC4) 1, transcript variant 2 | *MARCH1* | 2.93 |
| NM_024021 | membrane-spanning 4-domains, subfamily A, member 4, transcript variant 1 | *MS4A4A* | 2.93 |
| NM_005320 | histone cluster 1, H1d | *HIST1H1D* | 2.92 |
| NM_152304 | RAB42, member RAS oncogene family, transcript variant 2, | *RAB42* | 2.92 |
| NM_005994 | T-box 2 | *TBX2* | 2.92 |
| NM_000678 | adrenergic, alpha-1D-, receptor | *ADRA1D* | 2.91 |
| NM_173083 | lin-9 homolog (C. elegans) | *LIN9* | 2.91 |
| NM_013296 | G-protein signaling modulator 2 | *GPSM2* | 2.91 |
| NM_004526 | minichromosome maintenance complex component 2 | *MCM2* | 2.90 |
| NM_001182 | aldehyde dehydrogenase 7 family, member A1, nuclear gene encoding mitochondrial protein, transcript variant 1 | *ALDH7A1* | 2.88 |
| NM_000371 | transthyretin | *TTR* | 2.88 |
| NM_001129895 | hypothetical LOC100128124 | *HGC6.3* | 2.88 |
| NM_006207 | platelet-derived growth factor receptor-like | *PDGFRL* | 2.88 |
| NM_054034 | fibronectin 1, transcript variant 7 | *FN1* | 2.87 |
| NM_207437 | dynein, axonemal, heavy chain 10 | *DNAH10* | 2.87 |
| NM_017760 | non-SMC condensin II complex, subunit G2 | *NCAPG2* | 2.86 |
| NM_022909 | centromere protein H | *CENPH* | 2.86 |
| NM_152510 | HORMA domain containing 2 | *HORMAD2* | 2.86 |
| NM_032780 | transmembrane protein 25, transcript variant 1 | *TMEM25* | 2.85 |
| NM_022092 | CTF18, chromosome transmission fidelity factor 18 homolog (S. cerevisiae) | *CHTF18* | 2.84 |
| NM_031910 | C1q and tumor necrosis factor related protein 6, transcript variant 1 | *C1QTNF6* | 2.84 |
| NM_139314 | angiopoietin-like 4, transcript variant 1 | *ANGPTL4* | 2.83 |
| NM_000641 | interleukin 11 | *IL11* | 2.83 |
| NM_001333 | cathepsin L2, transcript variant 1 | *CTSL2* | 2.83 |
| NM_003012 | secreted frizzled-related protein 1 | *SFRP1* | 2.82 |
| NM_001733 | complement component 1, r subcomponent | *C1R* | 2.82 |
| NM_016269 | lymphoid enhancer-binding factor 1, transcript variant 1, | *LEF1* | 2.81 |
| NM_012339 | tetraspanin 15 | *TSPAN15* | 2.81 |
| NM_017805 | Ras interacting protein 1 | *RASIP1* | 2.81 |
| NM_153707 | family with sequence similarity 154, member A | *FAM154A* | 2.81 |
| NM_001007533 | dysferlin interacting protein 1 | *DYSFIP1* | 2.80 |
| NM_025135 | formin homology 2 domain containing 3 | *FHOD3* | 2.80 |
| NM_198270 | Nance-Horan syndrome (congenital cataracts and dental anomalies), transcript variant 1 | *NHS* | 2.80 |
| NM_024979 | MCF.2 cell line derived transforming sequence-like, transcript variant 2, | *MCF2L* | 2.79 |
| NM_001554 | cysteine-rich, angiogenic inducer, 61 | *CYR61* | 2.78 |
| NM_005431 | X-ray repair complementing defective repair in Chinese hamster cells 2 | *XRCC2* | 2.77 |
| NM_014288 | integrin beta 3 binding protein (beta3-endonexin), transcript variant 2 | *ITGB3BP* | 2.77 |
| NM_004111 | flap structure-specific endonuclease 1 | *FEN1* | 2.75 |
| NM_012338 | tetraspanin 12 | *TSPAN12* | 2.75 |
| NM_178835 | zinc finger protein 827 | *ZNF827* | 2.74 |
| NM_152524 | shugoshin-like 2 (S. pombe), transcript variant 1 | *SGOL2* | 2.74 |
| NM_014214 | inositol(myo)-1(or 4)-monophosphatase 2 | *IMPA2* | 2.73 |
| NM_003044 | solute carrier family 6 (neurotransmitter transporter, betaine/GABA), member 12, transcript variant 1 | *SLC6A12* | 2.73 |
| NM_016223 | protein kinase C and casein kinase substrate in neurons 3, transcript variant 2 | *PACSIN3* | 2.72 |
| NM_004370 | ollagen, type XII, alpha 1, transcript variant long | *COL12A1* | 2.72 |
| NM_002388 | inichromosome maintenance complex component 3 | *MCM3* | 2.72 |
| NM_020692 | UDP-N-acetyl-alpha-D-galactosamine:polypeptide N-acetylgalactosaminyltransferase-like 1, transcript variant 2 | *GALNTL1* | 2.71 |
| NM_025225 | patatin-like phospholipase domain containing 3 | *PNPLA3* | 2.71 |
| NM_198232 | ribonuclease, RNase A family, 1 (pancreatic), transcript variant 3, | *RNASE1* | 2.71 |
| NM_013282 | ubiquitin-like with PHD and ring finger domains 1, transcript variant 2 | *UHRF1* | 2.70 |
| NM_005496 | structural maintenance of chromosomes 4, transcript variant 1 | *SMC4* | 2.70 |
| NM_001105576 | ankyrin repeat domain 58 | *ANKRD58* | 2.69 |
| NM_003536 | histone cluster 1, H3h | *HIST1H3H* | 2.69 |
| NM_007268 | V-set and immunoglobulin domain containing 4, transcript variant 1 | *VSIG4* | 2.69 |
| NM_018132 | centromere protein Q | *CENPQ* | 2.69 |
| NM_006902 | paired related homeobox, transcript variant pmx-1a, | *PRRX1* | 2.68 |
| NM_005914 | minichromosome maintenance complex component 4, transcript variant 1 | *MCM4* | 2.68 |
| NM_001045556 | Src-like-adaptor, transcript variant 1 | *SLA* | 2.67 |
| NM_001101372 | IgLON family member 5 | *IGLON5* | 2.66 |
| NM_080685 | protein tyrosine phosphatase, non-receptor type 13 (APO-1/CD95 (Fas)-associated phosphatase), transcript variant 4, | *PTPN13* | 2.66 |
| NM_005654 | nuclear receptor subfamily 2, group F, member 1 | *NR2F1* | 2.65 |
| NM_001093 | acetyl-CoA carboxylase beta | *ACACB* | 2.63 |
| NM_032636 | proline/serine-rich coiled-coil 1, transcript variant 1 | *PSRC1* | 2.63 |
| NM_031479 | inhibin, beta E | *INHBE* | 2.63 |
| NM_004684 | SPARC-like 1 (hevin), transcript variant 2 | *SPARCL1* | 2.62 |
| NM_005951 | metallothionein 1H | *MT1H* | 2.61 |
| NM_013261 | peroxisome proliferator-activated receptor gamma, coactivator 1 alpha | *PPARGC1A* | 2.60 |
| NM_020995 | haptoglobin-related protein | *HPR* | 2.60 |
| NM_001012267 | centromere protein P | *CENPP* | 2.60 |
| NM_000043 | s Fas (TNF receptor superfamily, member 6), transcript variant 1 | *FAS* | 2.59 |
| NM_001003395 | tumor protein D52-like 1, transcript variant 2 | *TPD52L1* | 2.59 |
| NM_002129 | high mobility group box 2, transcript variant 1 | *HMGB2* | 2.59 |
| NM_001735 | complement component 5 | *C5* | 2.59 |
| NM_003782 | UDP-Gal:betaGlcNAc beta 1,3-galactosyltransferase, polypeptide 4 | *B3GALT4* | 2.58 |
| NM_003714 | stanniocalcin 2 | *STC2* | 2.57 |
| NM_004219 | pituitary tumor-transforming 1 | *PTTG1* | 2.56 |
| NM_178229 | IQ motif containing GTPase activating protein 3 | *IQGAP3* | 2.56 |
| NM_182647 | opiate receptor-like 1, transcript variant 1 | *OPRL1* | 2.56 |
| NM_182802 | minichromosome maintenance complex component 8, transcript variant 2 | *MCM8* | 2.55 |
| NR_028594 | zinc finger protein 833, pseudogene | *ZNF833P* | 2.55 |
| NM_001080434 | lemur tyrosine kinase 3 | *LMTK3* | 2.54 |
| NM_002030 | formyl peptide receptor 3 | *FPR3* | 2.54 |
| NM_002224 | inositol 1,4,5-trisphosphate receptor, type 3 | *ITPR3* | 2.54 |
| NM_024322 | centromere protein O, transcript variant 1 | *CENPO* | 2.54 |
| NM_001001547 | D36 molecule (thrombospondin receptor), transcript variant 2 | *CD36* | 2.53 |
| NM_000791 | dihydrofolate reductase | *DHFR* | 2.53 |
| NM_014985 | centrosomal protein 152kDa, transcript variant 2 | *CEP152* | 2.52 |
| NM_018451 | centromere protein J | *CENPJ* | 2.51 |
| NM_006607 | pituitary tumor-transforming 2 | *PTTG2* | 2.51 |
| NM_020190 | olfactomedin-like 3 | *OLFML3* | 2.51 |
